# On the Classification of ECG and EEG Signals with Various Degrees of Dimensionality Reduction

**DOI:** 10.3390/bios11050161

**Published:** 2021-05-19

**Authors:** Monica Fira, Hariton-Nicolae Costin, Liviu Goraș

**Affiliations:** 1 Institute of Computer Science, Romanian Academy, 700481 Iasi, Romania; monica.fira@iit.academiaromana-is.ro (M.F.); lgoras@etti.tuiasi.ro (L.G.); 2 Faculty of Medical Bioengineering, Grigore T. Popa University of Medicine and Pharmacy of Iasi, 700115 Iasi, Romania; 3 Faculty of Electronics, Telecomunications & Information Technology, Gheorghe Asachi Technical University of Iasi, 700050 Iasi, Romania

**Keywords:** dimensionality reduction, classifications, Laplacian eigenmaps, locality preserving projections, compressed sensing

## Abstract

Classification performances for some classes of electrocardiographic (ECG) and electroencephalographic (EEG) signals processed to dimensionality reduction with different degrees are investigated. Results got with various classification methods are given and discussed. So far we investigated three techniques for reducing dimensionality: Laplacian eigenmaps (LE), locality preserving projections (LPP) and compressed sensing (CS). The first two methods are related to manifold learning while the third addresses signal acquisition and reconstruction from random projections under the supposition of signal sparsity. Our aim is to evaluate the benefits and drawbacks of various methods and to find to what extent they can be considered remarkable. The assessment of the effect of dimensionality decrease was made by considering the classification rates for the processed biosignals in the new spaces. Besides, the classification accuracies of the initial input data were evaluated with respect to the corresponding accuracies in the new spaces using different classifiers.

## 1. Introduction

Manifold learning [1] is a method for reducing dimensionality using the fact that essential information for many classes of high dimensional signals lies in much smaller dimensional spaces/manifolds. This is as the process of generating the data happens to have fewer degrees of independence thus permitting to the transformed data to belong to a low-dimensional subspace. Thus, even though data can’t be represented in the initial space, when embedded in two or three dimensions, they can be easily represented and show, when possible some inherent structure. Therefore, to be able to visualize data dimension has to be decreased to one, two or three [2].

One possibility to get dimensionality reduction as well as compression is by taking projections of the data on a reduced number of random signals. However, using random projections, it is expected that some significant structure of the data might be lost since the signals are only approximately sparse and thus cannot be recovered with good accuracy [3].

Concerning geometry preserving, the techniques of manifold learning can be categorized into two classes:

(a) Techniques that preserve the local arrangement: locally linear embedding (LLE), Laplacian eigenmaps (LE), manifold charting (MC), Hessian locally linear embedding (HLLE), and
(b) Techniques that conserve global structure: isometric mapping (ISOMAP), diffusion map.

Several linear methods in manifold learning are principal component analysis (PCA), locality preserving projections (LPP) and multidimensional scaling (MDS), while among nonlinear ones are Isomap, Hessian eigenmaps, Laplacian eigenmaps, local linear embedding, and diffusion maps. From another point of view linear dimensionality reduction algorithms such as PCA, independent component analysis (ICA), linear discriminant analysis (LDA), and many others exhibit certain aspects to define an “interesting” way of linear data projection [4,5] at the price of possibly missing nonlinear structure of data. This is why non-linear methods are often stronger. The three steps of such algorithms are generally the following [6]:

- a nearest-neighbor search,
- defining of distances or affinities between elements,
- resolving a generalized eigenproblem to obtain the embedding of the initial space into a lower dimensional one.

The two main ingredients for dimensionality reduction are feature selection and feature extraction.

As mentioned above, we will discuss three methods for dimensionality reduction, two “standard” ones and the third, CS, which is not necessarily specific but interesting and useful as it will be shown.

In order to compare the methods we count on the fact that good dimensionality reduction will permit classification rates (usually smaller but) close to the initial ones.

We made use for testing, electrocardiographic (ECG) and electroencephalographic (EEG) signals downloaded from Internet databases and we compared the outcomes got with LE, LPP and CS using several standard classifiers aiming at getting an image about the compromise between dimensionality reduction and classification results.

In this paper we analyze the way the classifiers give good results for signals with various rates of dimensionality reduction. Thus, we present relevant information regarding the chosen method according to (a) the adopted rates of dimensionality reduction; (b) requirements such as reduced complexity (up to 2 or 3 dimensions), and (c) need for reconstruction. The advantages of each method are presented in the Section 4.

## 2. Materials and Methods

### 2.1. Laplacian Eigenmaps—LE

In the literature there are reported two similar techniques, in the sense that they consist each of three stages, the first two being common. The difference between the two is in the final stage, one of the algorithms keeping the local data arrangement, compared to the other that finds the optimal directions to project the data in a small space, so as to keep the data neighborhoods. These two techniques are Laplacian eigenmaps (LE) and locality preserving projections (LPP). Besides, for training data, Kernel LPP has the same significance as LE.

The basic assumption of the two methods is that data belong to a nonlinear subspace or nearly to it and in this way aim at discovering a low-dimensional modeling by retaining local characteristics. In LE the local properties are built on the keeping even distances between close neighbors.

The initial step in the LE algorithm [7] is to construct an adjacency graph G so that each data point xi is linked to its k nearest neighbors. In this way two things are important, namely, the number of neighbors as well as the weights of the graph branches which convey information about the distances between points.

The graph G will be constructed so that the weight wij is high if the points are close and wij is small if the nodes are far away. These weights are computed for all pairs of points xi and xj of the initial space; however, for points exterior the neighborhood k of a certain xm, the weights will have null value. In addition to the simplest weight assignment rule—one for neighboring points and null for outer points—a more exquisite rule is to use the Gaussian kernel [7,8,9]. After the calculation of the weights, follows the stage in which the calculation of the small dimensional representations is performed and on the manifold involves minimizing the cost function.

$$\emptyset \left(Y\right)=\sum_{\mathrm{ij}}∥y_{i}-y_{j}∥{}^{2}w_{\mathrm{ij}},$$

where great weights wij strongly penalize distant points, thus nearly items in the initial space will be represented as near as possible in the new low-dimensional space.

Briefly, the LE algorithm [9] can be sketched in three main steps, namely:

(i.) *Nearest-neighbor search and adjacency graph construction*

Choose a number between *K* or a distance ε > 0 such that the vicinities of each data point are established: for a *k*-neighborhood nodes *i* and *j* are linked by a branch if *i* is through the *k* nearest neighbors of *j* or *j* is through the *k* nearest neighbors of *i*. On the other hand, nodes *i* and *j* are linked by a branch if $∥x_{i}-x_{j}∥{}^{2}<\epsilon ,$ in which the Euclidean norm appears.

(ii.) *Weighted adjacency matrix (Choosing the weights)*

The weights *w_ij_* of the symmetric (*n × n*) vicinity matrix are computed as:

$$w_{\mathrm{ij}}=w\left(x_{i}-x_{j}\right)=\{\begin{array}{c} \exp\left\{-\frac{∥x_{i}-x_{\mathrm{ij}}∥{}^{2}}{2\sigma^{2}}\right\},\;if\;x\in N_{i};\\ \;\;\;\;\;\;0,\;otherwise, \end{array}$$

according to the graph *G* that is assumed to be connected.

(iii.) *Eigenmaps*

In this stage, the eigenvalues and eigenvectors are calculated for the general eigenvector problem,

Lf = λDf,(1)

where **D** = (d_ij_) is an (n × n) diagonal matrix with

$$d_{\mathrm{ii}}=\sum_{j\in N_{i}}w_{\mathrm{ij}},$$

and L = D − W is a Laplacian matrix which may be considered as an operator on functions applied on the nodes of *G*.

Ultimately, the eigenvector *f*_0_ suitable to the 0 eigenvalue is discarded. The next *m* eigenvectors related to the next *m* eigenvalues in increasing gamut are utilized for embedding in a *m*-dimensional Euclidean space:

x_i_ → (f_1_(i), …, f_m_(i)), (2)

where *f*_0_, …, *f_k−_*_1_ are the solutions of (1).

### 2.2. Locality Preserving Projections—LPP

The locality preserving projections (LPP) method is established on the similarly variation rule as for the LE method. It has alike locality conserving attributes: the training data are utilized to learn a projection and the testing samples are embedded into the low-dimensional space [10].

Therefore, the first two stages of the LPP algorithm are alike as those of the LE while the final stage assumes calculating the eigenvectors and eigenvalues for the generalized eigenvector problem:

XLXTa = λXDXTa,(3)

in which **X** is the training data matrix and L, D have the same meaning as before.

Designating with a_0_, …, a_l−1_ the column vectors related to the solutions of (2), ordering increasingly λ_0_ < … < λ_l-1_, the mapping is defined as:

(4) $$X_{i}\to y_{i}=A^{T}x_{I},\;A=\left(A_{0},{\;A}_{1},\ldots ,A_{l-1}\right),$$

in which *y_i_* is *l*-dimensional, and **A** is a (nxl) matrix.

### 2.3. Compressed Sensing—CS

Compressed sensing is an acquisition technique that requires fewer samples than the Nyquist rate in the hypothesis of sparsity of signals [11]. Thus a signal x can be expressed by the projections:

y = **∅** x, (5)

where $x\in R^{N}$, $y\in R^{M}$ is the projection vector and $\emptyset \in R_{M,\;N}$ is the compressed sensing matrix whose entries are random i.i.d. (independent and identically distributed) signals. In this paper we will use the low dimensional projection vector *y* for signal classifications [12] and not for restoration signals.

### 2.4. Classifier Types

Since there are many methods of classification presented in the literature, it is difficult to decide which algorithm is superior to the others. The choice of one or the other depends on the type of application in which the classifier is incorporated but also on the specifics of the type of data used in the application. For example, for the classes linear separable, if the classes are linearly separable, the linear classifiers as logistic regression, Fisher’s linear discriminant can surpass complex models as support vector machine (SVM) and artificial neural networks (ANN) and vice versa [13,14,15].

For the classification of ECG and EEG segments in the original space and in decreased dimensions, several classes of classifiers were used, namely: Decision Trees; Discriminant Analysis; Naive Bayes; SVM; Nearest Neighbor; Ensembles. Most of these classes have subclasses that have been used. In what follows several short descriptions of the main classifiers are given.

#### 2.4.1. Decision Trees

Given data of attributes annotated with classes, a decision tree provides a series of rules that can be applied to classify new data. It utilizes an *if-then* command set which is reciprocally exclusive and exhaustive for classification. The commands are read sequentially utilizing the training data one at a time. Each time a rule is learned, the tuples incorporated by the rules are eliminated. This process is sustained on the training set until fulfilling a finish condition.

Advantages: Decision Tree is easy to comprehend and to view, the data does not require much preparation and the method can manage both numerical and qualitative data.

Drawback: This method can yield trees that do not generalize well and can be unstable i.e., small fluctuations in data could lead to the generation of a completely different tree.

#### 2.4.2. Discriminant Analysis

This is a common primary classification method to test since it is quick, precise and simple to comprehend. Discriminant analysis is appropriate for voluminous datasets.

This technique presumes that particular categories provides data to whom they are assigned certain Gaussian distributions. In the training stage, the fitting function assesses the variables of a Gaussian law for every class.

#### 2.4.3. Naive Bayes

Bayes’ theorem is the source of this technique and it is based on the hypothesis of independence between every couple of attributes. Naive Bayes decision making behaves appropriately well in many real environments circumstances and applications, such as spam removal, document classification and person recognition. Naive Bayes is a simple method to apply and favorable outcomes have been acquired in the vast majority of situations. Additionally, it can be quickly used for voluminous datasets because it implies a linear function in time rather than by very time consuming iterative algorithms as in the case of a lot of other types of classifiers.

Advantages: Usually it needs a small number of training data to assess the necessary parameters. Naive Bayes decision making is very fast in contrast with more complex techniques.

Drawbacks: The big problem with this classifier is that it can manifest the so called “the zero probability problem”. Thus, in the situation where the conditioned probability is zero for a certain attribute, the classifier is not able to offer a correct decision. This problem is usually solved by means of a Laplacian estimator.

#### 2.4.4. Support Vector Machine—SVM

The support vector machine classifications consider the training data set as points divided into classes by an interval which is, ideally, as large as possible. The new data points are then embedded and estimated to belong to a certain class on one side or the other of the gap between the initial points.

In this way a SVM finds the most appropriate hyperplane that divides data points into two classes, in the sense that this hyperplane has the largest margin between the two classes. In other words, the SVM finds the maximal thickness of the area that is parallel to the hyperplane that has no inner data points [14].

Advantages: This classifier is efficient in high dimensional spaces and utilizes a subset of training data in the decision function that makes its memory very efficient.

Drawback: The SVM method does not directly give probability approximations. They are determined by applying usually an inefficient five-fold cross-validation.

#### 2.4.5. Nearest Neighbor

The neighbors based classification is a type of slow training as it does not attempt to build a universal internal pattern, but simply stores cases of the training data. Classification is estimated from a simple majority vote of the *k* nearest neighbors of each point. Upper bound of the error rate approaches twice that of the ideal Bayes classifier.

Benefits: This method is easy to apply, powerful for noisy training sets, and efficient if the training set is huge.

Drawback: The main problem is the necessity to calculate *k* and the computation effort is great as it needs to compute the distance of each input point to all the training data.

#### 2.4.6. Ensembles of Classifiers

The ensemble classifier combines a collection of classifiers that might perform superior classification performance compared to every single classifier. The principal rule behind the ensemble model is that a collection of poor learners join together to build a powerful learner. Qualities depend on the choice of the algorithm. Some techniques to perform ensemble decision trees are bagging and boosting.

Bagging (Bootstrap Aggregation) is applied when the object is to decrease the variance of a decision tree. The main idea is to create different data subsets from the training sample chosen randomly with replacement. Now, each group of subset data is utilized to train their decision trees. As a consequence, we end up with an ensemble of distinct models. Average of all the predictions from different trees are applied which is a more strong solution than a singular decision tree.

Boosting ensemble is another method to build a combination of classifiers. In this method, learners are determined sequentially with early learners applying uncomplicated models to the data and then evaluating data for errors. Hence, it fits consecutive trees (random sample) and, at all step, the object is to solve for net error from the previous tree.

Another type of ensemble of classifiers is the ensemble of nearest neighbor classifiers where each individual of the ensemble uses a random feature subset only and the decisions of these multiple classifiers are amalgamated for the ultimate decision.

Starting from the boosted trees ensemble, boosting being the most popular decision tree ensemble, Random under-sampling boosting (RUSBoost) has been introduced. Random under-sampling boosting (RUSBoost) is exceptionally successful at classifying irregular data. That means some classes with the training data have many more members than others. The method uses *N*, the number of members in the class with the fewest members in the training data, as the basic structure for sampling. In this way, by taking only *N* data points, classes with more members are under-sampled. If we have *K* classes, during the training stage, RUSBoost uses a smaller set of the data with *N* data points from each of those *K* classes. Then the method achieves the re-weighting and building the ensemble in Adaptive Boosting for Multiclass Classification [15].

## 3. Experimental Results and Discussions

### 3.1. ECG Signals

To analyze the feasibilities of dimension reduction utilizing LE, LPP and CS methods, we used for testing methods 44 ECG records from the MIT-BIH Arrhythmia database, including Holter data (so from wearable acquisition devices), collected at a sampling frequency of 360 Hz and on precision by 11 bits/sample [16]. Taking into account the annotations in the database, 7 pathological classes and the normal beating class were identified. The pathological classes included in this study are atrial premature beat (A), left bundle branch block beat (L), right bundle branch block beat (R), premature ventricular contraction (V), fusion of ventricular and normal beat (F), paced beat (/), fusion of paced and normal beat (f) and a class of normal beats (N).

**Figure 1:**
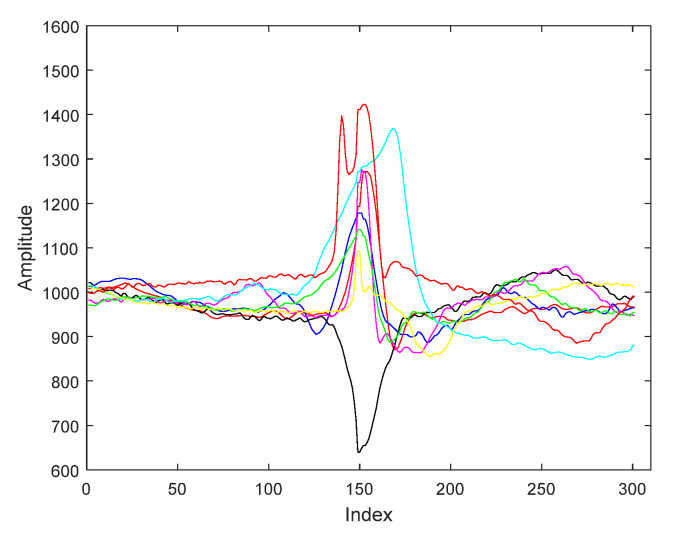
ECG patterns of the eight pattern classes used.

For segmentation ECG signals we applied the segmentation method presented in a previous paper, namely, segmentation with centered R wave [17]. Our segmentation method begins with the precise determination of the R-wave, which has the maximum amplitude of ECG. Thus, the ECG signals are split in heartbeats cycles. An ECG cycle starts in the midst of a certain RR interval and finishes in the midst of the following RR interval. The R wave is placed in the center of the ECG cycle by resampling the signals on both parts of R. Thus cycles with the centered R waveform have been computed. Thereby, all ECG cycles are defined by 301 samples with the R wave being situated on the 150-th sample. Figure 1 shows an example of segmentation of the ECG signals belonging to each of the eight pattern categories.

The database constructed is a data collection including 5608 ECG patterns, with 701 patterns for each of the eight considered types (seven pathological groups and a normal one).

A comparison of ECG behavior in the initial and reduced spaces implies first the classification of the ECG signals with the centered R-wave in the original space. The work was done in MATLAB^®^ medium (MathWorks, Natick, MA, USA) and we used the next classifiers, each with different versions for tuning their key settings: Decision Trees (with fine, medium and coarse type classifier), Linear Discriminant and Quadratic Discriminant, Naive and Kernel Naive Bayes, Support Vector Machine (Linear, Quadratic, Cubic and Gaussian), *k*-nearest neighbors (fine, medium, coarse, Cosine, Cubic and Weighted KNN), besides different kinds of the ensemble of classifiers (Boosted and Bagged trees, discriminant and KNN Subspace and RUSBoosted Trees).

**Figure 2:**
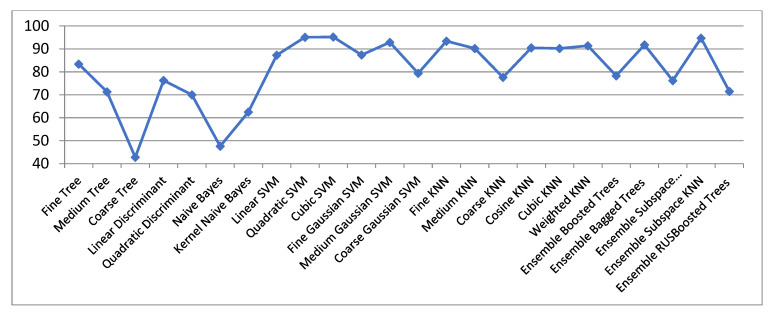
Classification rate in the original ECG space (centered 301 samples segments).

Figure 2 and Table 1 (its first column) show the classification accuracies for ECG signals with R-wave centered, in the initial space (raw data only). One can observe that good outcomes (over 90% classification accuracies) with SVM classifiers (Cubic, Quadratic and Medium Gaussian SVM), Fine KNN, and Ensemble Subspace KNN are got.

The decision borders obtained with the KNN classifier are much more complex than for all Decision Trees, so getting an excellent classification for Fine KNN. The bad outcomes got with Bayes as opposed to KNN may have the following explanation: the fundamental distinction between KNN and Naive Bayes methods is that KNN is a discriminative classifier, and the Naive Bayes is a generative classifier. The Fine KNN classifier behaves better because it has the characteristic to be optimized locally. The great results achieved with Fine KNN were expected to be so. With an ensemble subspace KNN even better outcomes may be acquired.

In our approach the best accuracy is achieved with Cubic SVM, i.e., 95.2%. This parameter is valuable because the 8 classes studied are not easily distinguishable, and they are even intertwining.

**Figure 3:**
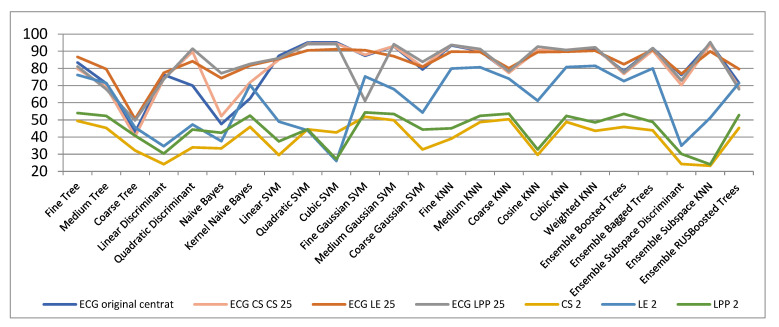
Classification results with CS, LE, LPP methods for 2, 3 and 25 dimensions, respectively.

In Table 1 and Figure 3 there are the classification outcomes: (a) in the original space with 301 samples; (b) results for ECG signals with dimensionality reduction by LE, LPP and CS methods for 2, 3 and 25 dimensions, respectively. We computed the classification accuracies for 2- and 3-dimensional cases because the signals with these dimensionalities can be easily illustrated graphically, which is very helpful and significant for comprehension the data spatial grouping. The graphic representation is very useful when we have many classes to handle and know nothing concerning their volumetric disposing. We also calculated the classification rate for dimensionality decrease to 25-space as we considered that a reduction from 301 to 25 dimensions is plausible both from the point of view of dimensionality reduction as well as in terms of classification accuracy.

**Figure 4:**
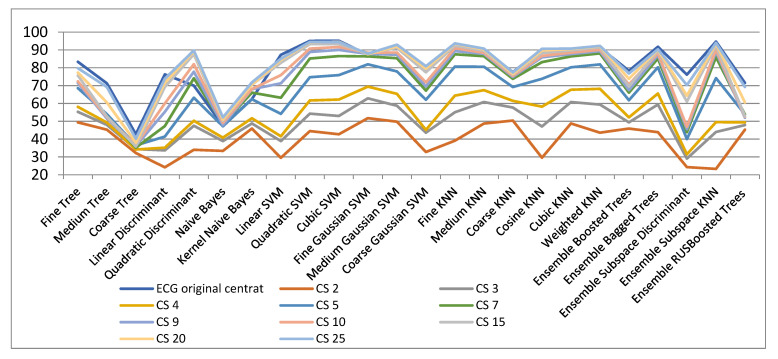
Classification results with CS method for dimensionality reduction.

Figure 4 and Table 2 show the results for various spatial dimensions for the Compressed Sensing (CS) method. It is observed that utilising Coarse Decision Tree very bad outcomes are got in the original space as well as in all other reduced spaces. Outcomes similar to those of the original space are achieved beginning with more than 10 dimensions in the projected space. Additionally, it can be observed the best outcomes hold with the SVM classifier. Depending on the degree of the dimensionality decrease they can be with cubic SVM or with fine Gaussian SVM. These classifiers achieve excellent classification rates, near to the medium Gaussian SVM. As a finding, for the dimensionality decrease with CS method, the SVM algorithm is best suited for that.

In the original 301-dimensional space the classification accuracy is 95.2%. In the case of decreasing to 10 and 25 dimensions, an accuracy of 91.7% and 93.4% were obtained, respectively. An interesting aspect that can be remarked in Table 2 (underlined numbers) is that for dimensionality reduction to 20 or 25 slightly improved results compared to those in the initial space have been obtained with some classifiers. A possible explanation is that through dimensionality reduction the classification problem complexity diminishes and thus the classification rate increases.

**Figure 5:**
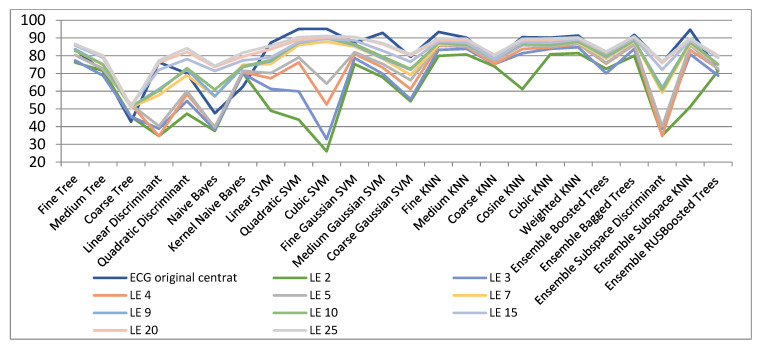
Classification results with LE method for dimensionality reduction.

Figure 5 and Table 3 show the results obtained with LE, both for the initial and reduced ECG signals. In the original space the best outcomes are attained with cubic SVM classifier. On the contrary, in the case of very small dimensions (between 2 and 5) of the projected space with the LE algorithm very weak outcomes are achieved. For very small manifolds, the best outcomes are accomplished with the Weighted KNN classifier. This statement can be justified by maintaining the vicinities at the local level. Likewise, excellent outcomes for very small spaces are obtained by using the Fine Gaussian SVM classifier. Thus, for these small spaces, the classification of the test data is strongly dependant on the quality of the classifier. In other words, the classifier has to be able to draw very precise decision limits for very close data. It is the case of the Fine Gaussian SVM kernel range, that is establish to (1/4) sqrt(no. of features).

However, the Laplacian Eigenmaps technique for very small spaces, such as 2 and 3 dimensions, leads to very good classification results (81.5% and 84.5% classification accuracy, respectively) with Weighted KNN classifier. It is to remember here that the current classification problem is a difficult one, as there are 8 categories of ECG signals. We may state that a classification rate with only almost 10% under the original space versus a decrease in size from 301 to 2 is a remarkable result. The exceptional benefit of shrinking to 2 or 3 dimensions is the input data may be easily visualized graphically, allowing certain comprehension of the spatial arrangement. For a dimensionality reduction over 10, it can be observed that for some classifiers (results underlined in Table 3) higher classification accuracy than in the initial space has been obtained reminding of a kind of feature selection algorithm.

**Figure 6:**
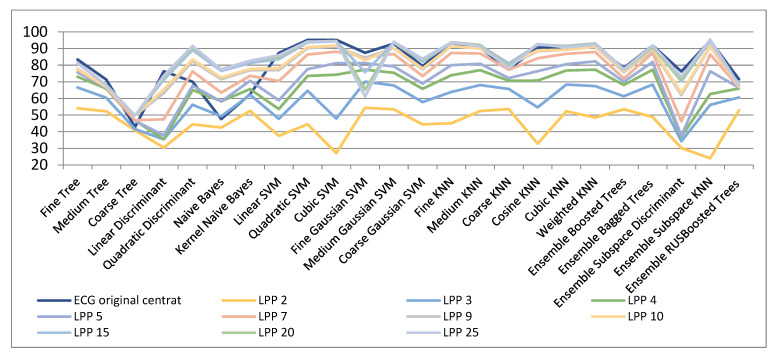
Classification results with LPP method for dimensionality reduction.

Figure 6 and Table 4 show the results of dimensionality reduction when using the LPP algorithm. As seen, the results are very similar to those achieved with the Laplacian Eigenmaps technique besides for very low dimensions (of 2, 3, and 4), when the classification measures achieved are much inferior (54%, 70.1%, and 77.3%, respectively). In the case of dimensions superior to 5, the classification measures are similar to those attained with the Laplacian Eigenmaps technique. For dimensions upper 20, classification measures very near to those in the original space are reached. As an example, for 20- and 25-dimensional spaces classification accuracies of above 95% are achieved by means of the Ensemble Subspace KNN classifier.

It has been observed again (underlined numbers in Table 4) that for dimensionality reduction over 10, in some cases improved results have been obtained.

**Figure 7:**
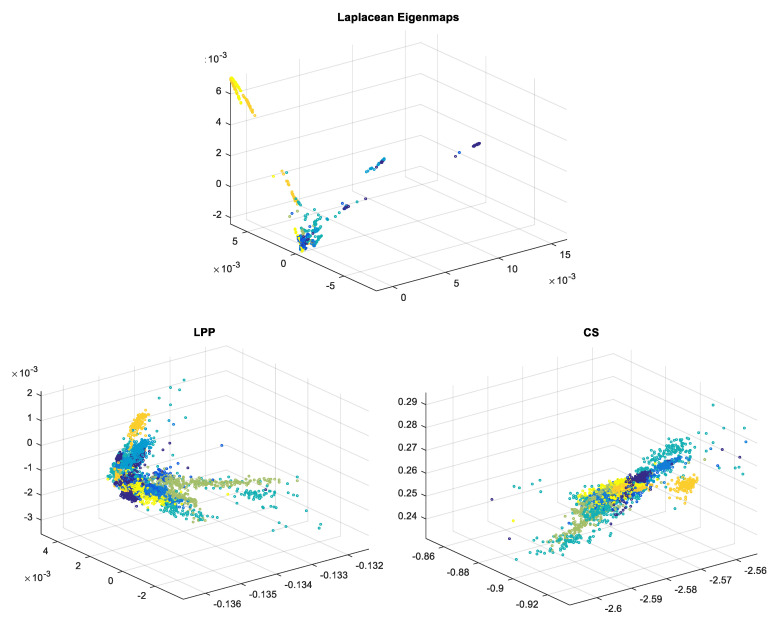
ECG data mapped into a 3-dimensional space with LE, LPP and CS techniques.

In Figure 7 ECG signals with reduced dimensionality to 3D obtained with the 3 techniques are presented (each color corresponds to a different class) [18]; the great advantage of the possibility of data graphical visualization is obvious.

It can be observed that LE leads to a better data clustering/spatial separation than the other two methods for which, even though data are clustered, overlapping occurs. This is the reason why, when choosing dimensionality reduction to 3D, the classification ratio is better for LE compared to LPP and CS.

### 3.2. EEG Signals

For testing the dimensionality reduction methods, the EEG signals collected by Hoffmann and collaborators in their laboratory were used; a small database is free on the internet at [19]. This database includes EEG signals collected on the configuration with 32 channels, arranged in 942 vectors to be classified, lasting 1 sec. each [20,21]. The classification task is to detect the P300 waveform from a single EEG trial which has been used to build a P300 based spelling device for Brain-Computer Interface—BCI. We used configurations with 23, 8 and 4 channels for original EEGs for preprocessing and classifications tasks. The paradigm with P300 spelling device [22] that has been used is as follows.

One of the first examples for BCI is the algorithm proposed by Farwell and Donchin [22] that relies on the unconscious decision-making processes expressed via P300 in order to lead a computer. Another example, described in [23], refers to a real-time training of voted perceptron for classification of EEG data, also for a BCI application.

**Figure 8:**
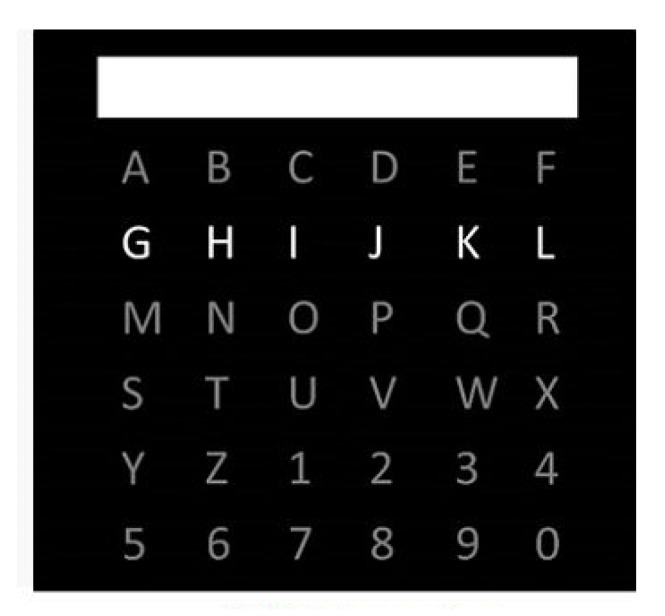
Classical P300 spelling paradigm described by Farwell–Donchin (1988).

Now returning to the experiments proposed in [22], a (6 × 6) matrix containing (as in Figure 8) the letters of the alphabet and the numbers 1–9 were shown to the subjects on a computer display. The horizontal and vertical lines of the table were run at random for 100 ms with a 100 ms pause between sparkles i.e., after 12 sparkles every horizontal and vertical line was glowing once. Two datasets were acquired from every subject. During the first meeting subjects were requested to write the French words “lac”, “nuage”, “montagne”, and “soleil”, while for the second recording the subjects had to write the words “fromage”, “chocolat”, “pain”, and “vin” [21].

As reported in [20] the EEG signals were registered from channels FP1, FP2, AF3, AF4, F7, F3, FZ, F4, F8, FC1, FC5, FC6, FC2, T7, C3, CZ, C4, T8, CP1, CP5, CP6, CP2, P7, P3, PZ, P4, P8, PO3, PO4, O1, OZ, O2 with a Biosemi Active 2 system (NEUROSPEC AG, Stans, Switzerland) at 2048 Hz. The signals were then referred to the average of channels O1, OZ, O2, low pass filtered (0…9) Hz with a 7th order Butterworth filter, and re-sampled with 128 Hz. The channels used as reference and channels T7, T8 were not used for EEG processing as they did not bring significant information for the P300s waveform detection. A more detailed explanation of the experimental work, i.e., EEG acquisition, preprocessing and artifact rejection is presented in [21].

**Figure 9:**
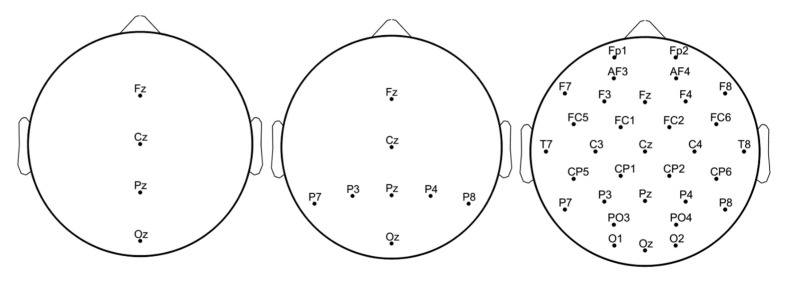
The electrodes configurations with 4, 8 and 23 channels.

In Figure 9 the electrodes configurations with 4, 8 and 23 channels are shown.

**Figure 10:**
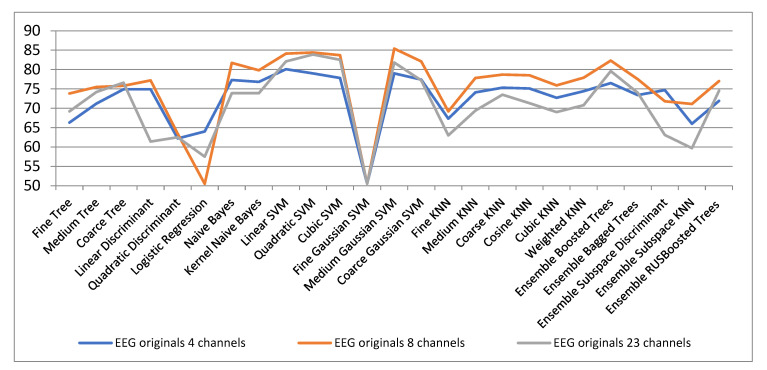
Classification results with original EEG signals for configurations with 4, 8 and 23 channels.

Figure 10 shows the classification results for different channel configurations cases. It is observed that in general for the 8-channel version the best classification results of the original EEG signals are obtained. In general, good results are obtained for linear, quadratic and cubic SVM, but the best results are obtained with medium Gaussian SVM in the 8-channel configuration.

Because, in general, the configuration with 8 electrodes offers the best results, in the following we will present the results of this configuration for dimensionality reduction through the three analyzed methods. It should be mentioned that the initial EEG signals are segmented according to the stimulus applied to segments of 128 samples, i.e., we will consider that the space of the initial EEG signals is 128-dimensional.

**Figure 11:**
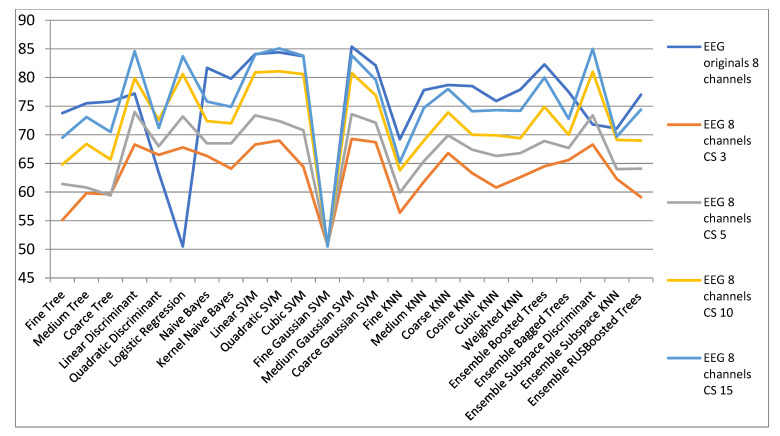
Results for the dimensionality reduction with CS algorithm for configurations with 8 channels.

Figure 11 and Table 5 show the results for the dimensionality reduction with CS algorithm. It is found that there are classifiers with which better results are obtained in a space reduced to 15 dimensions compared to the initial space. This is the case of the discriminant linear classifier for which in the original space the classification rate is 77.2% and in a space reduced to 15 dimensions it classifies with a rate of 84.6%. Additionally, Quadratic Discriminant and Logistic Regression offers improved results for all spaces compared to the initial space. Additionally, in the case of Discriminant Subspace Ensembles the results in the reduced spaces are generally superior to the initial space. These results for which in spaces of reduced dimensionality improved results are obtained, compared to the initial spaces, are an example that the initial signals are in reality in a space of a much smaller dimensionality. It is much easier to classify data with a small dimension compared to the same data that is represented in a false large space.

**Figure 12:**
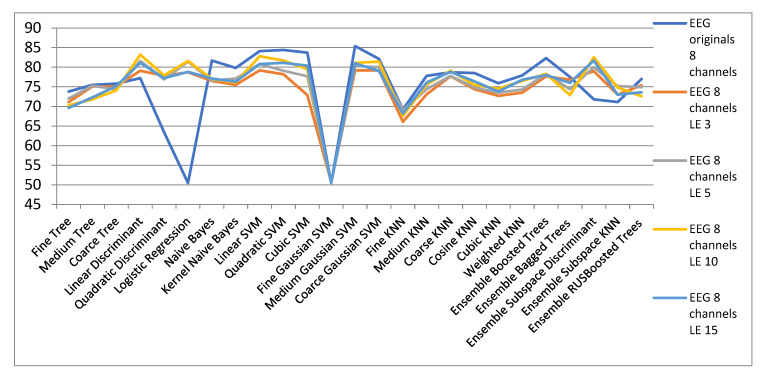
Results for the dimensionality reduction with LE algorithm for configurations with 8 channels.

Figure 12 shows the results obtained with the LE algorithm to reduce the dimensionality of the space for EEG signals in the 8-channel configuration. It can be seen in Table 6 that in the case of the CS algorithm, the Linear and Quadratic Discriminant and Logistic Regression classifiers offer improved classification rates. Additionally, Discriminant Subspace Ensembles and KNN Subspace Ensembles classify better in reduced spaces with LE algorithm. The major difference from the CS method is that for very small spaces of dimensionality 3 and 5 the results are much better for the LE method compared to CS method. Hence the utility of the LE algorithm for data representation in 2 and 3 dimensional spaces for better visualization and understanding of spatial and geometric data arrangement.

**Figure 13:**
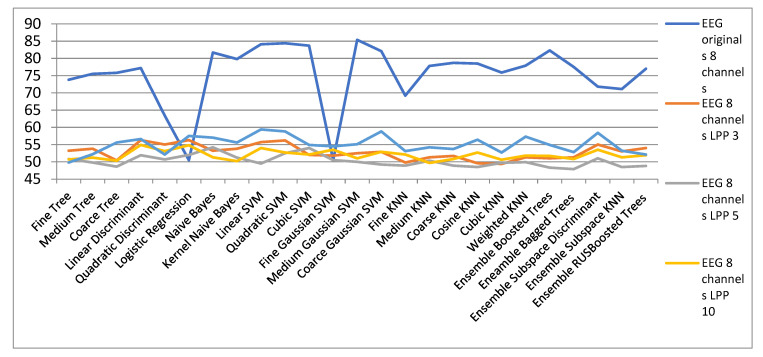
Results for dimensionality reduction with LPP algorithm for configurations with 8 channels.

Figure 13 shows the results obtained with the LPP algorithm to reduce the dimensionality of space for EEG signals in the 8-channel configuration. It is observed in Table 7 that the best results are obtained with all the classifiers for the initial space. These poor results are obtained both when applying LPP on each channel and then concatenating the signals with small spaces, or concatenating the initial EEG signals for the 8 channels and then applying the LPP method for dimensionality reduction.

**Figure 14:**
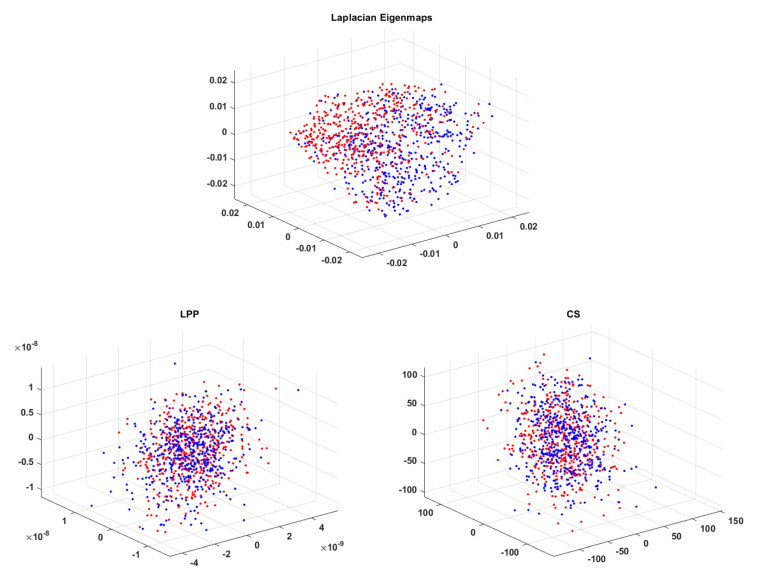
EEG data mapped into a 3-dimensional space with LE, LPP and CS techniques.

In Figure 14 EEG signals with dimensionality reduced to 3D with all three techniques are represented. Signals containing the P300 wave have been plotted in blue and the others in red. It can be observed that for CS and LPP the two classes overlap, thus explaining the modest classification results for the 3D case. When using LE we get a better clustering of the two classes on the left laying non-P300 waves marked in red and on the right the P300 ones marked in blue. This is why LE leads to better results for 3D compared to LPP and CS.

## 4. Conclusions

The aim of the paper was to offer a general view of the way the classifiers give good results for signals with various rates of dimensionality reduction.

Regarding ECG signals we stress the fact that they were preprocessed by aligning the R-wave. Our best results were obtained with SVM and KNN while for low dimensions (2 or 3), the best outcomes have been achieved with LE with the drawback that computations should be repeated for any new signal. Additionally, it has been found that in the case of CS for more than 10 dimensions the classification rate is near that obtained in the original space. Similar classification rates results have been achieved for dimensionality reduction larger than 10 with LPP for which the advantage for new testing signal is that no new calculations are necessary. Regarding CS, it is the most computationally advantageous compared to LE and LPP, which are much more computationally expensive.

For EEG signals, the CS and LE algorithms led to results similar to those obtained for ECG signals. The major difference that occurs in the case of EEG signals is for the LPP algorithm. This leads to much weaker results in reducing the dimensionality of the signals. To explain these results, we propose two hypotheses. A first one is that the LPP algorithm cannot find universal optimal projections for all 8 channels. The second hypothesis is that in the case of EEG signals the data are located on a manifold and the LPP algorithm fails to capture the local and at the same time general structure of the manifold, a situation encountered, for example, in the Swiss Roll manifold case.

The main conclusions of this work envisage the way dimensionality reduction and classification algorithms can be combined in order to obtain reasonable classification results even for (very) low dimensions both for ECG and a class of EEG signals. Choosing the rate of reduction of dimensionality is dependent on the motivation of the analysis. Thus, if we intend to reconstruct the initial signal, we will adopt CS, if we want intuition for 2 or 3 D we will choose LE while if we want to reduce dimensionality by about ten–twelve times and make classification in the reduced space without re-computation for new signals, we will use LPP. However, it seems LPP does not fit too well the global structure for EEG signals so that between LPP and LE the second one is better.

We assume these methods and outcomes might be extended in specific limits for more types of signals too, yet this concept should be attentively applied.

## Figures and Tables

**Table 1:** Classification accuracies with CS, LE, LPP algorithms for 2, 3 and 25 dimensions respectively.

	ECG Original Centered	Compressed Sensed (CS)			Laplacian Eigenmaps (LE)			Locality Preserving Projections (LPP)		
	ECG Original	CS 2	CS 3	CS 25	LE 2	LE 3	LE 25	LPP 2	LPP 3	LPP 25
Fine Trees	83.44	49.41	55.34	79.81	76.25	77.32	86.73	54.00	66.65	81.15
Medium Trees	71.32	45.35	48.00	69.23	71.53	68.85	79.62	52.34	60.43	67.91
Coarse Trees	42.83	32.21	34.41	40.32	45.64	45.64	50.67	40.85	41.54	49.75
Linear Discriminant	76.32	24.23	33.72	73.94	34.77	38.81	77.44	30.42	35.41	73.64
Quadratic Discriminant	70.00	34.00	47.53	89.77	47.34	54.54	84.22	44.41	56.24	91.51
Naive Bayes	47.63	33.43	38.93	52.22	37.64	38.34	74.36	42.51	49.37	77.21
Kernel Naive Bayes	62.53	45.94	48.8	71.85	70.34	69.95	81.74	52.54	62.26	82.64
Linear SVM	87.34	29.52	38.9	85.14	49.08	61.37	85.62	37.52	47.72	85.92
Quadratic SVM	95.11	44.54	54.3	94.54	43.95	59.92	90.54	44.52	64.64	94.24
Cubic SVM	**95.24**	42.72	53.00	**94.50**	26.10	33.00	**91.20**	27.10	47.92	94.24
Fine Gaussian SVM	87.47	**51.80**	**62.90**	87.91	75.36	78.75	90.69	**54.40**	**70.10**	61.14
Medium Gaussian SVM	92.91	49.84	58.74	93.00	67.92	69.88	87.12	53.44	67.84	94.14
Coarse Gaussian SVM	79.47	32.85	43.65	80.97	54.36	55.41	80.92	44.45	57.82	83.82
Fine KNN	93.42	39.14	55.14	93.71	79.92	83.36	89.84	45.11	63.90	93.74
Medium KNN	90.27	48.72	60.82	90.82	80.76	83.92	89.65	52.42	68.00	91.32
Coarse KNN	77.62	50.47	57.71	77.44	74.00	75.35	80.12	53.63	65.74	78.34
Cosine KNN	90.54	29.64	47.15	90.74	61.25	81.42	89.55	32.80	54.62	92.76
Cubic KNN	90.22	48.81	60.81	90.81	80.88	83.95	89.72	52.38	68.34	90.77
Weighted KNN	91.47	43.60	59.44	92.34	**81.52**	**84.82**	90.32	48.51	67.42	92.35
Ensemble Boosted Trees	78.34	45.97	49.45	76.81	72.65	70.19	82.49	53.55	61.36	77.67
Ensemble Bagged Trees	91.81	43.94	59.45	90.4	80.00	83.91	90.91	48.86	68.31	91.84
Ensemble Subspace Discriminant	76.24	24.31	29.14	70.3	35	38.95	76.93	30.22	34.32	73.05
Ensemble Subspace KNN	94.71	23.34	44.00	94.04	51.24	80.82	89.98	24.14	56.10	**95.34**
Ensemble RUS Boosted Trees	71.54	45.34	47.94	69.31	71.54	68.84	79.64	52.84	60.67	67.97

**Table 2:** Classification results with CS method for dimensionality reduction.

	ECG Original Centered	CS 2	CS 3	CS 4	CS 5	CS 7	CS 9	CS 10	CS 15	CS 20	CS 25
Fine Tree	83.4	49.4	55.3	58.1	68.6	72.3	71.5	72.4	75.7	77.3	79.8
Medium Tree	71.3	45.3	48.0	49.3	54	52.8	51.6	52.3	52.7	60.6	69.2
Coarse Tree	42.8	32.2	34.4	34.2	36.5	35.2	36.2	36.7	35.9	38.0	40.3
Linear Discriminant	76.3	24.2	33.7	35.2	41.4	47.3	55.3	60.0	69.2	71.6	73.9
Quadratic Discriminant	70.0	34.0	47.5	50.3	63.2	74.1	77.8	82.0	87.6	*89.1*	*89.7*
Naive Bayes	47.6	33.4	38.9	40.8	47.2	48.6	47.8	49.1	50.3	50.9	*52.2*
Kernel Naive Bayes	62.5	45.9	48.8	51.7	62.4	*66.1*	*68.0*	*68.1*	*70.5*	*70.5*	*71.8*
Linear SVM	87.3	29.5	38.9	41.6	54.2	63.2	71.3	75.9	82.8	84.4	85.1
Quadratic SVM	95.1	44.5	54.3	61.7	74.7	85.2	88.9	90.8	93.3	94.2	94.5
Cubic SVM	**95.2**	42.7	53.0	62.2	75.9	86.6	**90.1**	**91.7**	**93.4**	**94.7**	**94.5**
Fine Gaussian SVM	87.4	**51.8**	**62.9**	**69.5**	**82.0**	86.4	*87.8*	*88.5*	*88.0*	*87.6*	*87.9*
Medium Gaussian SVM	92.9	49.8	58.7	65.4	78.0	85.4	87.3	88.6	91.2	92.0	*93.0*
Coarse Gaussian SVM	79.4	32.8	43.6	45.2	62.1	67.2	69.5	71.8	77.5	*79.5*	*80.9*
Fine KNN	93.4	39.1	55.1	64.4	80.7	87.6	89.4	91.0	92.4	*93.5*	*93.7*
Medium KNN	90.2	48.7	60.8	67.5	80.6	86.5	87.8	88.4	89.6	*90.3*	*90.8*
Coarse KNN	77.6	50.4	57.7	61.5	69.2	73.8	74.9	75.5	76.3	76.6	77.4
Cosine KNN	90.5	29.6	47.1	58.2	73.8	83.2	85.9	86.7	88.3	89.7	90.7
Cubic KNN	90.2	48.8	60.8	67.7	80.3	86.4	87.7	88.5	89.8	*90.5*	*90.8*
Weighted KNN	91.4	43.6	59.4	68.2	81.9	**88.1**	89.3	90.1	91.5	*92.1*	*92.3*
Ensemble Boosted Trees	78.3	45.9	49.4	52.2	61.8	66.1	67.5	70.6	69.5	73.8	76.8
Ensemble Bagged Trees	91.8	43.9	59.4	65.6	80.3	85.2	87.1	88.2	89.7	90.2	90.4
Ensemble Subspace Discriminant	76.2	24.3	29.1	31.5	40.0	43.9	45.6	47.0	61.1	64.4	70.3
Ensemble Subspace KNN	94.7	23.3	44.0	49.5	74.2	86.0	89.0	90.3	92.4	93.6	94.0
Ensemble RUSBoosted Trees	71.5	45.3	47.9	49.4	53.9	53.8	52.0	52.5	52.8	60.6	69.3

**Table 3:** Classification results with LE method for dimensionality reduction.

	ECG Original Centred	LE 2	LE 3	LE 4	LE 5	LE 7	LE 9	LE 10	LE 15	LE 20	LE 25
Fine Tree	83.4	76.2	77.3	80.4	80.4	82.9	*83.7*	*82.8*	*85.8*	*86.5*	*86.7*
Medium Tree	71.3	71.5	68.8	*72.7*	*72.4*	*74.9*	*75*	*75.1*	*78.9*	*80.1*	*79.6*
Coarse Tree	42.8	*45.6*	*45.6*	*52.5*	*52.5*	*50.9*	*51.2*	*51.3*	*51.8*	*51.6*	*50.6*
Linear Discriminant	76.3	34.7	38.8	34.7	40.3	57.8	61.1	60.3	72.1	76.2	77.4
Quadratic Discriminant	70	47.3	54.5	58.3	60.1	69	*72.2*	*73*	*78.1*	*82.1*	*84.2*
Naive Bayes	47.6	37.6	38.3	39.8	39.5	*57*	*57.1*	*60.9*	*71.4*	*73.7*	*74.3*
Kernel Naive Bayes	62.5	*70.3*	*69.9*	*70.8*	*71.5*	*74.9*	*73.6*	*74*	*77.3*	*79.5*	*81.7*
Linear SVM	87.3	49	61.3	67.3	70.2	75.3	76.9	77.5	79.1	83.7	85.6
Quadratic SVM	95.1	43.9	59.9	76.2	79	86.1	87.6	87.3	87.7	89	90.5
Cubic SVM	**95.2**	26.1	33	52.5	64.2	87.9	**90.1**	**89.7**	89.6	90.4	**91.2**
Fine Gaussian SVM	87.4	75.3	78.7	81.1	82	85.2	85.9	86.5	*88.6*	*90.4*	*90.6*
Medium Gaussian SVM	92.9	67.9	69.8	73.4	75.4	78.3	78.6	79.5	82.8	86.6	87.1
Coarse Gaussian SVM	79.4	54.3	55.4	61.2	66.2	69.2	72.1	72.5	76.6	*80.1*	*80.9*
Fine KNN	93.4	79.9	83.3	85.7	86.2	86.2	87.2	87.1	88.1	88.9	89.8
Medium KNN	90.2	80.7	83.9	85	85.5	86.8	87	86.3	87.4	88.9	89.6
Coarse KNN	77.6	74	75.3	75.3	77.1	*79*	*78.6*	*78.5*	*78.3*	*80.6*	*80.1*
Cosine KNN	90.5	61.2	81.4	83.8	85.9	86.9	86.7	86.9	87.6	88.9	89.5
Cubic KNN	90.2	80.8	83.9	84.7	85.5	86.8	86.8	86.1	87.4	89	89.7
Weighted KNN	91.4	**81.5**	**84.8**	**86.6**	**86.9**	87.4	88.1	87.8	89.1	89.9	90.3
Ensemble Boosted Trees	78.3	72.6	70.1	75.5	76	78.3	79.2	79.9	*81.4*	*82.2*	*82.4*
Ensemble Bagged Trees	91.8	80	83.9	86.2	86.6	**88.2**	88.6	88.7	**89.9**	**90.9**	90.9
Ensemble Subspace Discriminant	76.2	35	38.9	34.7	40.2	59.2	61.9	60.5	72.2	75.9	76.9
Ensemble Subspace KNN	94.7	51.2	80.8	83.2	86.1	86.9	87.6	87.8	88.7	89.6	89.9
Ensemble RUSBoosted Trees	71.5	71.5	68.8	*72.7*	*72.4*	*74.9*	*75*	*75.1*	*79*	*80.1*	*79.6*

**Table 4:** Classification results with LPP method for dimensionality reduction.

	ECG Original Centered	LPP 2	LPP 3	LPP 4	LPP 5	LPP 7	LPP 9	LPP 10	LPP 15	LPP 20	LPP 25
Fine Tree	83.4	54	66.6	73	75.6	77.2	77.8	77.5	81.5	81.3	81.1
Medium Tree	71.3	52.3	60.4	65.9	66.5	66.8	66.9	67	68	68.1	67.9
Coarse Tree	42.8	40.8	41.5	*46.7*	*46.6*	*46.9*	*49.7*	*49.9*	*49.7*	*49.7*	*49.7*
Linear Discriminant	76.3	30.4	35.4	35.5	37.8	47.5	63.2	65.3	71.2	72.6	73.6
Quadratic Discriminant	70	44.4	56.2	65.1	67.6	*76.2*	*82.3*	*83.4*	*89.1*	*90.5*	*91.5*
Naive Bayes	47.6	42.5	49.3	*58.3*	*58.1*	*63.5*	*71.5*	*72.5*	*76.5*	*77.5*	*77.2*
Kernel Naive Bayes	62.5	52.5	62.2	*65.6*	*70.6*	*73.6*	*77*	*77.7*	*81.3*	*82.6*	*82.6*
Linear SVM	87.3	37.5	47.7	53.6	58.9	70.4	76.9	78.1	83.5	84.8	85.9
Quadratic SVM	95.1	44.5	64.6	73.5	77.6	86.4	90.2	90.9	93.7	94.1	94.2
Cubic SVM	**95.2**	27.1	47.9	74.3	81.2	**88.1**	91.2	**91.8**	94.3	94.5	94.2
Fine Gaussian SVM	87.4	**54.4**	**70.1**	**77.3**	81.2	84.8	84.4	82.9	75.8	65.2	61.1
Medium Gaussian SVM	92.9	53.4	67.8	75.4	79.2	86.7	90.2	90.4	*93.5*	*93.8*	*94.1*
Coarse Gaussian SVM	79.4	44.4	57.8	65.8	68.9	73.4	77.4	78	*82.1*	*83*	*83.8*
Fine KNN	93.4	45.1	63.9	73.9	80	87.3	**91.4**	91.5	*93.3*	*93.8*	*93.7*
Medium KNN	90.2	52.4	68	77	80.8	87	89.9	89.9	*91.9*	*92.1*	*91.3*
Coarse KNN	77.6	53.6	65.7	70.6	72.2	77.3	*80*	*80.3*	*81*	*79.3*	*78.3*
Cosine KNN	90.5	32.8	54.6	70.7	76.4	84.1	88.4	88.9	*92.2*	*92.7*	*92.7*
Cubic KNN	90.2	52.3	68.3	76.8	80.6	86.8	89.2	89.3	*91.6*	*91.1*	*90.7*
Weighted KNN	91.4	48.5	67.4	**77.3**	**82.3**	87.9	*91*	*91.1*	*93*	*92.9*	*92.3*
Ensemble Boosted Trees	78.3	53.5	61.3	68.1	70	72	75.8	76.5	77.6	77.3	77.6
Ensemble Bagged Trees	91.8	48.8	68.3	77.2	81.9	87.3	89.1	89.9	91.2	90.8	91.8
Ensemble Subspace Discriminant	76.2	30.2	34.3	37	37.7	46.3	62	63.2	70.3	70.9	73
Ensemble Subspace KNN	94.7	24.1	56.1	62.6	76.3	86.4	91.2	91.6	**94.5**	**95.4**	**95.3**
Ensemble RUSBoosted Trees	71.5	52.8	60.6	66	66.5	66.8	66.8	67.1	68	68.1	67.9

**Table 5:** Classification results with CS method for configurations with 8 channels.

	ECG Orig.	EEG 8 Channels CS			
	8 Channels	CS 3	CS 5	CS 10	CS 15
Fine Tree	73.8	55.1	61.4	64.8	69.5
Medium Tree	75.5	59.8	60.8	68.4	73.1
Coarse Tree	75.8	59.6	59.4	65.7	70.5
Linear Discriminant	77.2	68.3	74	79.9	84.6
Quadratic Discriminant	63.4	66.5	68	72.6	71.2
Logistic Regression	50.5	67.8	73.2	80.6	83.7
Naive Bayes	81.7	66.3	68.5	72.4	75.8
Kernel Naive Bayes	79.8	64.1	68.5	72	74.9
Linear SVM	84.1	68.3	**73.4**	80.9	84
Quadratic SVM	84.4	69	72.4	**81.1**	**85.1**
Cubic SVM	83.7	64.4	70.8	80.6	83.8
Fine Gaussian SVM	50.5	50.7	50.5	50.5	50.5
Medium Gaussian SVM	**85.4**	**69.3**	73.6	80.8	83.9
Coarse Gaussian SVM	82.1	68.7	72.1	76.9	79.6
Fine KNN	69.2	56.4	59.9	63.8	65.2
Medium KNN	77.8	61.8	65.4	69	74.7
Coarse KNN	78.7	66.8	69.9	73.9	78
Cosine KNN	78.5	63.3	67.4	70	74.1
Cubic KNN	75.9	60.8	66.3	69.9	74.3
Weighted KNN	77.9	62.6	66.8	69.4	74.2
Ensemble Boosted Trees	82.3	64.5	68.9	74.9	80
Ensemble Bagged Trees	77.5	65.6	67.7	70	72.8
Ensemble Subspace Discriminant	71.8	68.3	73.4	81	85
Ensemble Subspace KNN	71.1	62.3	64	69.1	69.7
Ensemble RUSBoosted Trees	77	59.1	64.1	69	74.4

**Table 6:** Classification results with LE algorithm for configurations with 8 channels.

	ECG Originals	EEG 8 Channels LE			
	8 Channels	LE 3	LE 5	LE 10	LE 15
Fine Tree	73.8	71.1	72	70.3	69.6
Medium Tree	75.5	75.1	75.3	71.8	72.3
Coarse Tree	75.8	75.1	74.3	74.1	75.2
Linear Discriminant	77.2	79.1	**81.6**	**83.2**	81.1
Quadratic Discriminant	63.4	77.8	76.9	77.9	77.2
Logistic Regression	50.5	78.7	81.4	81.6	78.8
Naive Bayes	81.7	76.5	76.6	77	77.1
Kernel Naive Bayes	79.8	75.5	77.1	76.1	76.3
Linear SVM	84.1	**79.2**	80.8	82.8	80.8
Quadratic SVM	84.4	78.2	79.1	81.7	81.1
Cubic SVM	83.7	72.9	77.7	79.5	80.4
Fine Gaussian SVM	50.5	50.7	50.5	50.5	50.5
Medium Gaussian SVM	**85.4**	**79.2**	80.3	81.1	81
Coarse Gaussian SVM	82.1	**79.2**	80	81.4	79
Fine KNN	69.2	66.1	69.1	67.6	68.2
Medium KNN	77.8	73.1	74.4	75.6	76.1
Coarse KNN	78.7	77.7	77.8	79.1	78.8
Cosine KNN	78.5	74.4	74.8	75.5	76.4
Cubic KNN	75.9	72.7	73.5	74.5	73.8
Weighted KNN	77.9	73.5	74.3	76.5	76.8
Ensemble Boosted Trees	82.3	77.7	78.3	78.3	78
Ensemble Bagged Trees	77.5	76.8	74.4	72.9	76
Ensemble Subspace Discriminant	71.8	79	80	82.5	**81.7**
Ensemble Subspace KNN	71.1	73	75.2	74.8	73
Ensemble RUSBoosted Trees	77	75.4	74.9	72.6	73.6

**Table 7:** Classification results with LPP algorithm for configurations with 8 channels.

	EEG Orig.	EEG 8 Channels			
	8 Channels	LPP 3	LPP 5	LPP 10	LPP 15
Fine Tree	73.8	53.2	50.8	50.7	49.8
Medium Tree	75.5	53.8	49.8	51.2	52.2
Coarse Tree	75.8	50.4	48.6	50.3	55.6
Linear Discriminant	77.2	**56.3**	51.9	**54.9**	56.6
Quadratic Discriminant	63.4	55	50.7	53.1	52.1
Logistic Regression	50.5	**56.3**	52	**54.8**	57.5
Naïve Bayes	81.7	53.2	**54.2**	51.3	57
Kernel Naïve Bayes	79.8	53.8	51.2	50.2	55.6
Linear SVM	84.1	55.7	49.5	54	**59.4**
Quadratic SVM	84.4	56.2	52.5	52.7	58.8
Cubic SVM	83.7	52	54	52.1	54.9
Fine Gaussian SVM	50.5	51.8	50.5	53.5	54.5
Medium Gaussian SVM	**85.4**	52.5	50	51	55.1
Coarce Gaussian SVM	82.1	52.9	49.2	52.9	58.8
Fine KNN	69.2	49.8	48.9	52.1	53.1
Medium KNN	77.8	51.3	50.3	49.7	54.2
Coarse KNN	78.7	51.7	48.9	50.8	53.7
Cosine KNN	78.5	49.6	48.5	52.7	56.4
Cubic KNN	75.9	49.4	49.7	50.6	52.7
Weighted KNN	77.9	51.3	49.9	51.8	57.3
Ensemble Boosted Trees	82.3	51	48.3	51.7	54.9
Ensemble Bagged Trees	77.5	51.3	47.9	50.8	52.8
Ensemble Subspace Discriminant	71.8	55	51	53.5	58.4
Ensemble Subspace KNN	71.1	53	48.5	51.3	53.2
Ensemble RUSBoosted Trees	77	54	48.8	51.9	52.1

## Data Availability

The data presented in this study are openly available in [physionet] at [10.1109/51.932724 and 10.1161/01.cir.101.23.e215], reference number [16] and [epfl], reference number [19]. The webpage of the MIT-BIH Arrhythmia Database is “https://www.physionet.org/content/mitdb/1.0.0/” (accessed on 17 May 2021) and “http://mmspg.epfl.ch/cms/page-58322.html” (accessed on 22 May 2017).
